# INTERACTION OF RESILIENCE AND DISTRESS IN THE ELDERLY-CAREGIVER DYADS: AN ACTOR-PARTNER INTERDEPENDENCE MODEL

**DOI:** 10.1093/geroni/igad104.3437

**Published:** 2023-12-21

**Authors:** Anni Wang, zhiyao xiong

**Affiliations:** Fudan University, Shanghai, Shanghai, China (People’s Republic); Huaxi Hospital, Chengdu, Sichuan, China (People’s Republic)

## Abstract

**Aims:**

The aim of this study was to explore the interaction between resilience and psychological distress in disabled elderly-caregiver dyads. Design: A cross-sectional descriptive study.

**Methods:**

A total of 246 homebound disabled elderly individuals and their family caregivers from five provinces of China were investigated by convenience sampling using the Chinese version of the Resilience Scale and the Distress Thermometer and Problem List. Data analysis was conducted using the actor-partner interdependence model (APIM) method and based on structural equation modelling.

**Results:**

The effect of resilience on psychological distress shows a mixed pattern. With regard to the actor effect, the resilience of disabled elderly individuals and caregivers was negatively related to their own psychological distress. Regarding the partner effect, the resilience of disabled elderly individuals was negatively associated with caregiver psychological distress, and the resilience of the caregiver was negatively associated with the psychological distress of the disabled elderly individual.

**Conclusion:**

There is a dyadic interaction between disabled elderly individuals and their caregivers, suggesting that resilience can relieve not only one’s own psychological stress but also that of others to some extent. Health professionals can design dyadic interventions that focus on resilience and its dyadic interaction and effectively leverage the resilience advantages of the dyad based on the individuals’ level of psychological distress to improve the mental health of elderly people with disabilities and their caregivers.

